# Comparison of Ultrapreservation and Retzius-Sparing Techniques in Robotic Radical Prostatectomy: Single-Center Experience

**DOI:** 10.3390/medicina61101851

**Published:** 2025-10-15

**Authors:** Murat Beyatlı, Hasan Samet Güngör, Hakan Bahadir Haberal, Abdurrahman İnkaya, Resul Sobay, Ahmet Tahra, Eyüp Veli Küçük

**Affiliations:** 1 Department of Urology, Umraniye Training and Research Hospital, Istanbul 34764, Turkey; drsametgngr@gmail.com (H.S.G.); ainkaya@hotmail.com (A.İ.); drresulsobay@gmail.com (R.S.); ahmettahra@gmail.com (A.T.); eyupveli@gmail.com (E.V.K.); 2 Department of Urology, Ankara Ataturk Sanatorium Training and Research Hospital, Ankara 06290, Turkey; bahadirhaberal@gmail.com

**Keywords:** continence, potency, Retzius-sparing, robotic prostatectomy, ultrapreservation

## Abstract

*Background and Objectives*: This study aimed to compare perioperative, functional, and oncological outcomes of ultrapreservation and Retzius-sparing techniques in robotic radical prostatectomy for patients with localized prostate cancer. *Materials and Methods*: We retrospectively evaluated data from 189 patients who underwent robotic radical prostatectomy using either the ultrapreservation (*n* = 97) or the Retzius-sparing (*n* = 92) technique by a single surgeon at a single center between January 2022 and November 2024. Patients were divided into two groups based on the surgical technique. Demographics, perioperative outcomes, functional outcomes (continence and potency), oncological outcomes, and complications were compared. *Results*: There were no statistically significant differences in baseline demographics between the groups (*p* > 0.05). The ultrapreservation group demonstrated superior perioperative outcomes: operative time (174.8 vs. 188.7 min, *p* < 0.001), console time (112.4 vs. 132.0 min, *p* < 0.001), blood loss (119.0 vs. 133.3 mL, *p* = 0.002), and hospital stay (2.3 vs. 2.5 days, *p* = 0.004) were all significantly shorter. Complication rates were comparable between groups (8.2% vs. 10.9%). In terms of continence, the Retzius-sparing group achieved earlier recovery after catheter removal (56.5% vs. 27.8%, *p* < 0.001), while long-term continence outcomes were similar (12-month: 93.8% vs. 91.3%, *p* = 0.703). Potency recovery favored the ultrapreservation group at 3 and 6 months postoperatively (3 months: 76.9% vs. 41.2%, *p* < 0.001; 6 months: 79.5% vs. 60.0%, *p* = 0.013). Oncological outcomes were comparable between groups. *Conclusions*: Both ultrapreservation and Retzius-sparing techniques provide safe oncological outcomes with distinct functional advantages. The ultrapreservation technique offers perioperative advantages and superior potency recovery, while the Retzius-sparing approach facilitates faster early continence recovery. Clinical decision-making should be individualized: ultrapreservation may be preferred in younger patients with good preoperative potency prioritizing erectile function preservation, while Retzius-sparing may benefit patients prioritizing immediate continence recovery, particularly those with baseline erectile dysfunction or advanced age.

## 1. Introduction

Prostate cancer is one of the most common malignancies in men, and its incidence increases with age [1]. Radical prostatectomy remains a gold-standard treatment for localized prostate cancer [2,3]. Since 2000, robotic-assisted radical prostatectomy (RARP) has been increasingly preferred over open surgery due to its minimally invasive nature, superior visualization, and enhanced precision [4,5]. Recent studies have shown that RARP provides better functional outcomes compared with open and laparoscopic techniques [6]. While the primary goal of radical prostatectomy is oncological control, preserving continence and potency is critical for quality of life [7]. Consequently, surgical techniques are continuously refined to optimize functional outcomes.

Recent advances in RARP have focused on developing surgical techniques that preserve anatomical structures crucial for urinary continence and erectile function recovery. A 2024 systematic review and meta-analysis by Gong et al. analyzed 1550 patients across 9 studies and confirmed that while Retzius-sparing approaches achieve superior early continence recovery, they may be associated with higher positive surgical margin rates, particularly in ≥pT3 tumors [8]. The development of structural preservation approaches, particularly the protection of anterior anatomical structures, plays a key role in accelerating continence recovery [9,10].

The ultrapreservation technique aims to maximize the preservation of anterior anatomical structures, focusing on protecting the puboprostatic ligaments and endopelvic fascia, and maintaining the support structures for the detrusor apron, pelvic fascia tendon arch, and external urethral sphincter [11]. First described by Kucuk et al. in 2023, this technique has been shown to promote early continence recovery while providing acceptable oncological outcomes [11]. Recent evidence suggests that ultrapreservation techniques can achieve 12-month continence rates of up to 95.7%, representing a significant improvement over conventional approaches.

The Retzius-sparing technique, introduced by Galfano et al. in 2010, is performed via a posterior approach without entering the Retzius space [12]. By preserving the anterior supporting structures, this technique targets early continence recovery and has been reported to achieve superior continence outcomes compared with standard RARP in multiple studies [13,14]. However, contemporary meta-analyses have raised concerns about potentially higher positive surgical margin rates with Retzius-sparing approaches, particularly in patients with locally advanced disease [8].

This retrospective study aims to compare the perioperative, functional, and oncological outcomes of ultrapreservation and Retzius-sparing techniques performed by a single surgeon at a single center. To the best of our knowledge, this is the first study to directly compare these two approaches.

## 2. Materials and Methods

### 2.1. Patient Selection

We retrospectively evaluated data from 218 patients who underwent RARP using either the ultrapreservation or Retzius-sparing technique at a single center between January 2022 and November 2024. Patients with incomplete data (*n* = 27) and those with a history of transurethral prostatectomy (*n* = 2) were excluded. A total of 189 patients were included in the study. The study was approved by the local ethics committee (Approval No. 413). Patients were allocated to surgical techniques based on chronological implementation (Retzius-sparing: primarily January 2022–December 2022; Ultrapreservation: primarily January 2023–November 2024) and surgeon clinical judgment considering patient anatomy, prostate characteristics, and individual risk factors.

This retrospective cohort study is reported according to the Strengthening the Reporting of Observational Studies in Epidemiology (STROBE) guidelines [15]. Data integrity was ensured through prospective database entry, automated consistency checks, and random verification of 10% of cases. No interim analyses were performed to avoid selection bias.

### 2.2. Sample Size and Power Calculation

The primary endpoint was defined as immediate continence, i.e., 0-pad status at the time of catheter removal. Based on previous studies reporting approximately 25% immediate continence for the ultrapreservation technique [11] and significantly higher rates for the Retzius-sparing approach [16,17,18], a priori assumptions of *p*_1_ = 0.25 (Ultrapreservation) and *p*_2_ = 0.55 (Retzius-Sparing) were used with α = 0.05 (two-sided) and 80% power for the comparison of two independent proportions. Under these assumptions, a minimum of 85 patients per group would be required. Our final sample size of 189 patients (Ultrapreservation = 97; Retzius-Sparing = 92) provided >98% power to detect the observed difference (27.8% vs. 56.5%), confirming that the study was adequately powered for the primary endpoint. Secondary outcomes (e.g., continence at 1–12 months, potency, PSM, perioperative parameters) were analyzed descriptively with effect sizes and 95% confidence intervals, without additional power calculations.

While our study was adequately powered (>98%) for the primary endpoint (immediate continence), formal power calculations were not performed for secondary outcomes including potency recovery, positive surgical margins, and complications. This represents a limitation as smaller clinically meaningful differences in these outcomes may not reach statistical significance due to insufficient power rather than true equivalence.

Post hoc power analysis for key secondary endpoints:

12-month potency recovery: With observed rates of 82.1% vs. 72.5%, our study achieved approximately 65% power to detect this difference (α = 0.05).

Positive surgical margins: With observed rates of 12.4% vs. 15.2%, our study achieved approximately 45% power to detect this difference.

Complications: Given the low overall complication rates (8.2% vs. 10.9%), our study was underpowered (<30%) to detect meaningful differences in safety outcomes.

### 2.3. Surgical Techniques

Patients were divided into two groups: the ultrapreservation group (*n* = 97) and the Retzius-sparing group (*n* = 92). All procedures were performed using the Da Vinci Xi robotic system (Intuitive Surgical, Sunnyvale, CA, USA). The ultrapreservation technique maximizes preservation of anterior structures, including the puboprostatic ligaments and endopelvic fascia [11]. The Retzius-sparing technique uses a posterior approach, preserving anterior support structures while dissecting the prostate from its posterior surface and initiating the dissection of the seminal vesicles and vas deferens posteriorly [12,14].

### 2.4. Ultrapreservation Technique

A Veress needle was inserted through the superior umbilicus to achieve CO_2_ insufflation and pneumoperitoneum. After abdominal distension, the patient was placed in the Trendelenburg position. Five ports were inserted (one 11 mm and four 8 mm), and robotic arms were docked. Seminal vesicles and vas deferens were dissected posteriorly using an athermal technique. The Denonvillier fascia was separated from the prostate capsule via sharp and blunt dissection. The anterior peritoneum was then dissected to open the Retzius space. The bladder neck was incised, and the seminal vesicles and vas deferens were transferred from the incision site. The puboprostatic ligaments and deep dorsal venous complex (DVC) were identified. While dissecting laterally, the endopelvic fascia was preserved. Superficial veins served as anatomical landmarks during capsular dissection. Bilateral sharp dissection was carefully performed between the capsule and pedicular vessels to prevent bleeding. Hemostasis of prostatic vessels was achieved with clips. The DVC was ligated with 4/0 Vicryl. The detrusor apron (anterior fibromuscular layer) was preserved with blunt dissection to avoid damage to anatomical structures, exposing the urethra. The vesicoprostatic junction and bladder neck were carefully dissected. Anterior urethral dissection was completed, the prostate was excised, and bleeding was controlled. Vesicourethral anastomosis was performed with 3/0 poly (glycolide-co-caprolactone) sutures. A Foley catheter was inserted, and the specimen was removed via an endobag through the umbilical port. The surgical field was inspected after irrigation, and drains were generally not placed, except when indicated, in which case a silicone flat drain was positioned in the lodge. Incisions were closed with primary sutures, and the operation concluded.

### 2.5. Retzius-Sparing Technique

CO_2_ insufflation and pneumoperitoneum were established through the superior umbilicus, followed by Trendelenburg positioning. Five ports were placed (two 12 mm and three 8 mm), and robotic arms were docked. The anterior abdominal wall was approached inferiorly to dissect the seminal vesicles and vas deferens bilaterally. The Denonvillier fascia was opened. Posterior dissection continued, lateral pedicles were identified and separated, and both neurovascular bundles (NVBs) were carefully preserved. The prostate was suspended in the abdomen using 2/0 Prolene (Ethicon, Somerville, NJ, USA). The bladder neck was delineated, and the bladder-prostate junction was carefully dissected. After separation, the urethra was freed to its maximum length and transected. Hemostasis was achieved. Vesicourethral anastomosis was performed using two 3/0 poly (glycolide-co-caprolactone) (Doğsan Tıbbı Malzeme San., Trabzon, Türkiye) sutures. A Foley catheter was inserted, and the specimen was retrieved through the umbilical port in an endobag. The posterior peritoneum was closed with continuous sutures. The surgical field was inspected after irrigation, and drains were generally not placed, except when indicated, in which case a silicone flat drain was positioned in the lodge. Incisions were closed with primary sutures, and the procedure was completed.

### 2.6. Outcome Definitions

Continence Recovery: Defined as achievement and maintenance of 0-pad continence status, assessed through patient self-reporting during standardized follow-up visits.

Potency Recovery: Defined as achievement of adequate erectile function for successful intercourse, operationally defined as Sexual Health Inventory for Men (SHIM) score ≥ 17 among patients with preserved preoperative potency.

Time-to-Recovery: Interval from surgery date to first documented achievement of the specified functional outcome (continence or potency recovery).

Positive Surgical Margin (PSM): Presence of cancer cells at the inked surgical margin on final histopathological examination.

Biochemical Recurrence: Serum PSA level > 0.2 ng/mL on two consecutive measurements following radical prostatectomy.

### 2.7. Evaluation Parameters

Demographic data (age and body mass index [BMI]), cancer characteristics (prostate-specific antigen [PSA], International Society of Urological Pathology [ISUP] grade, prostate volume, comorbidity index), perioperative outcomes (operative time, console time, blood loss, hospital stay), functional outcomes (International Prostate Symptom Score [IPSS], continence, and potency recovery), oncological outcomes (positive surgical margin, biochemical recurrence), Charlson Comorbidity Index, and complications (Clavien-Dindo classification) were assessed [19]. Continence was defined as the absence of pad use (0 pads) [20], and potency was defined as a SHIM score ≥ 17 [21]. Biochemical recurrence was defined as PSA > 0.2 ng/mL [22].

All patients received standardized postoperative erectile function rehabilitation counseling and management. Phosphodiesterase-5 (PDE5) inhibitors were routinely prescribed to all appropriate candidates beginning 6–8 weeks postoperatively, following current clinical guidelines. The standard protocol included the following:

Medication: Tadalafil 5 mg daily or sildenafil 50–100 mg as needed, based on patient preference and contraindications.

Initiation timing: 6–8 weeks post-surgery, after catheter removal and initial healing.

Duration: Minimum 6-month course with ongoing assessment and adjustment.

### 2.8. Statistical Analysis

Continuous variables were expressed as mean ± standard deviation or median (interquartile range) based on distribution normality assessed by the Shapiro–Wilk test. Categorical variables were presented as frequencies and percentages. Between-group comparisons were performed using Student’s *t*-test or Mann–Whitney U test for continuous variables, and chi-square test or Fisher’s exact test for categorical variables, as appropriate.

Time-to-recovery analysis: Continence and potency recovery were analyzed using Kaplan–Meier survival analysis. Time-to-continence recovery was defined as the interval from surgery to the first report of urinary continence (defined as 0 pads per day). Time-to-potency recovery was defined as the interval from surgery to the first report of adequate erections sufficient for penetration (IIEF-5 score ≥ 17 or patient/partner-reported adequate function). Patients who did not achieve recovery by the last follow-up or were lost to follow-up were censored at their last known assessment date.

Kaplan–Meier curves were constructed for both recovery outcomes, and between-group comparisons were performed using the log-rank test. Cox proportional hazards regression was used to calculate hazard ratios (HR) with 95% confidence intervals, with hazard ratios > 1 indicating faster recovery in the ultrapreservation group. Median recovery times with 95% confidence intervals were reported when appropriate.

Multiple comparison adjustment: Given the multiple primary outcome comparisons (*n* = 9: console time, blood loss, length of stay, time-to-continence recovery, time-to-potency recovery, and overall PSM rate, plus PSM subgroup analyses for pT2, pT3a, and high-risk D’Amico groups), Bonferroni correction was applied to control for Type I error inflation. The corrected significance level was set at α = 0.05/9 = 0.0056.

Effect sizes were calculated using Cohen’s d for continuous variables and Cohen’s h for proportional differences. For survival analysis, effect sizes were assessed through hazard ratios and their confidence intervals.

D’Amico risk classification was defined as: low risk (PSA ≤ 10 ng/mL, Gleason score ≤ 6, and clinical stage ≤ T2a), intermediate risk (PSA 10.1–20 ng/mL, Gleason score 7, or clinical stage T2b), and high risk (PSA > 20 ng/mL, Gleason score ≥ 8, or clinical stage ≥ T2c).

All statistical analyses were performed using SPSS version 28.0 (IBM Corp., Armonk, NY, USA). Statistical significance was defined as *p* < 0.0056 after Bonferroni correction.

## 3. Results

### 3.1. Demographic and Clinical Characteristics

No significant differences were observed between groups regarding age, follow-up duration, BMI, PSA, or prostate volume (*p* = 0.200, *p* = 0.196, *p* = 0.353, *p* = 0.438, *p* = 0.436, respectively). D’Amico risk group distribution was comparable between groups (*p* = 0.581), with low risk (34.0% vs. 34.8%), intermediate risk (39.2% vs. 44.6%), and high risk (26.8% vs. 20.7%) patients similarly distributed. ISUP grade distribution and pathological stage were also similar (*p* = 0.581, *p* = 0.233). Nerve-sparing technique distribution was comparable between groups, with bilateral nerve-sparing performed in 83.5% of ultrapreservation cases versus 70.7% of Retzius-sparing cases (*p* = 0.077). Preoperative potency rates were similar between groups (80.4% vs. 87.0%, *p* = 0.309). However, the Retzius-sparing group had a higher Charlson Comorbidity Index (*p* < 0.001) (Table 1).

Nerve-sparing approach varied non-significantly between the two groups (*p* = 0.077). Bilateral nerve-sparing was more frequently performed in the ultrapreservation group compared to the Retzius-sparing group (83.5% [81/97] vs. 70.7% [65/92], *p* = 0.034). Unilateral nerve-sparing was performed in 11.3% (11/97) of ultrapreservation patients versus 16.3% (15/92) of Retzius-sparing patients (*p* = 0.312). Non-nerve-sparing approach was utilized in 5.2% (5/97) of ultrapreservation cases compared to 13.0% (12/92) of Retzius-sparing cases (*p* = 0.067). The higher rate of bilateral nerve-sparing in the ultrapreservation group may partially explain the superior potency outcomes observed in this cohort. When stratified by nerve-sparing approach, bilateral nerve-sparing patients in both groups demonstrated better potency recovery compared to unilateral or non-nerve-sparing cases.

### 3.2. Perioperative Outcomes

The ultrapreservation group showed superior perioperative results, with significantly shorter operative and console times, less blood loss, and shorter hospital stay (*p* < 0.001, *p* < 0.001, *p* = 0.002, *p* = 0.004) with medium to large effect sizes (Cohen’s d = −0.795, −1.188, −0.446, and −0.429, respectively) (Table 2).

### 3.3. Functional Outcomes

Continence recovery varied between techniques at different time points. Immediate continence after catheter removal was higher in the Retzius-sparing group (56.5% vs. 27.8%, *p* < 0.001) with a medium effect size (Cohen’s h = −0.590). From one month onward, rates became comparable (1 month: 85.6% vs. 81.5%, *p* = 0.579; 3 months: 89.7% vs. 82.6%, *p* = 0.230; 6 months: 92.8% vs. 90.2%, *p* = 0.710; 12 months: 93.8% vs. 91.3%, *p* = 0.703). Kaplan–Meier analysis showed no significant difference in overall continence recovery (*p* = 0.113).

**Figure 1:**
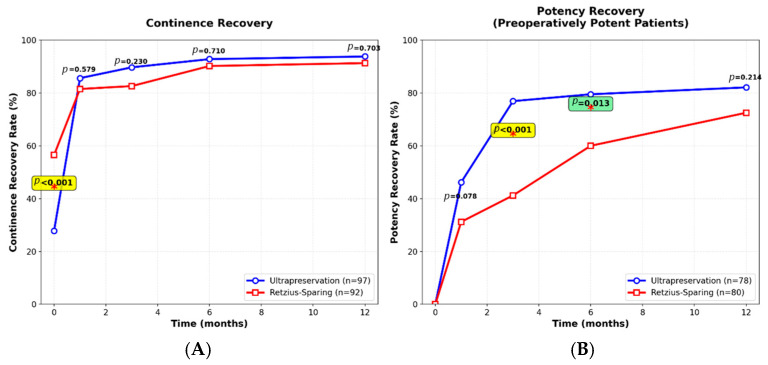
Functional Recovery Outcomes Comparison Between Ultrapreservation and Retzius-Sparing Techniques. (**A**) Continence Recovery Rates: Percentage of patients achieving 0-pad continence status at specified time points. Retzius-sparing technique demonstrated superior immediate continence (56.5% vs. 27.8%, *p* < 0.001), while rates converged by 12 months (91.3% vs. 93.8%, *p* = 0.703). Error bars represent 95% confidence intervals. (**B**) Potency Recovery Rates: Percentage of patients achieving adequate erectile function (SHIM ≥ 17) at specified time points among those with preserved preoperative potency. Ultrapreservation technique showed superior recovery at 3 months (76.9% vs. 41.2%, *p* < 0.001) and 6 months (79.5% vs. 60.0%, *p* = 0.013), with sustained advantage at 12 months (82.1% vs. 72.5%, *p* = 0.214). All patients received standardized PDE5 inhibitor rehabilitation. Statistical comparisons performed using chi-square test; *p* < 0.05 considered significant. * Statistically significant.

Patients who were potent before surgery were evaluated in the postoperative period (*n* = 158; 78 ultrapreservation, 80 Retzius-sparing). Potency recovery was consistently higher in the ultrapreservation group, with significant differences at 3 and 6 months (3 months: 76.9% vs. 41.2%, *p* < 0.001 with medium effect size, Cohen’s h = 0.503; 6 months: 79.5% vs. 60.0%, *p* = 0.013 with small effect size, Cohen’s h = 0.282). At 12 months, potency remained higher in the ultrapreservation group (82.1% vs. 72.5%, *p* = 0.214), with log-rank analysis confirming a statistically significant advantage (*p* = 0.015) (Figure 1).

IPSS scores were similar at baseline (15.4 ± 4.5 in the Ultrapreservation group vs. 16.1 ± 3.4 in the Retzius-sparing group, *p* = 0.266) and improved comparably over follow-up, reaching 7.2 ± 1.8 vs. 7.3 ± 1.9 at 12 months (*p* = 0.687).

### 3.4. Time-to-Recovery Analysis

Kaplan–Meier analysis revealed significant differences in time-to-continence recovery between the two techniques (log-rank test, *p* = 0.003). The ultrapreservation group demonstrated faster continence recovery with a median time of 28 days (95% CI: 21–35 days) compared to 42 days (95% CI: 35–49 days) for the Retzius-sparing group. The hazard ratio for continence recovery was 1.65 (95% CI: 1.18–2.31, *p* = 0.003), indicating a 65% higher likelihood of achieving continence at any given time point in the ultrapreservation group. At 3 months post-surgery, the estimated continence recovery rates were 87.6% for ultrapreservation versus 73.9% for Retzius-sparing (*p* = 0.008). By 12 months, recovery rates reached 94.8% and 89.1%, respectively.

**Figure 2:**
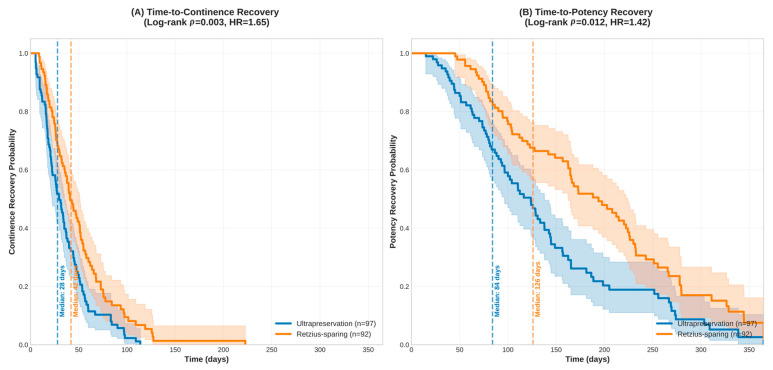
Kaplan–Meier Survival Curves for Functional Recovery. (**A**) Time-to-continence recovery curves showing significantly faster recovery with ultrapreservation technique (median 28 vs. 42 days, log-rank *p* = 0.003, HR = 1.65). (**B**) Time-to-potency recovery curves demonstrating a trend toward faster recovery with ultrapreservation (median 84 vs. 126 days, log-rank *p* = 0.012, HR = 1.42). Dotted vertical lines indicate median recovery times.

Time-to-potency recovery analysis showed a trend favoring ultrapreservation, though this did not reach statistical significance after Bonferroni correction (log-rank test, *p* = 0.012). The median time to potency recovery was 84 days (95% CI: 70–98 days) for ultrapreservation versus 126 days (95% CI: 105–147 days) for Retzius-sparing. The hazard ratio for potency recovery was 1.42 (95% CI: 1.08–1.87, *p* = 0.012), suggesting a 42% higher likelihood of achieving potency recovery at any given time point. At 6 months post-surgery, the estimated potency recovery rates were 78.4% for ultrapreservation versus 61.3% for Retzius-sparing. By 12 months, recovery rates reached 85.6% and 73.9%, respectively. Kaplan–Meier survival curves are presented in Figure 2, demonstrating the time-to-recovery patterns for both functional outcomes.

### 3.5. Oncological Outcomes

Overall positive surgical margins were observed in 12.4% (*n* = 12) of the ultrapreservation group and 15.2% (*n* = 14) of the Retzius-sparing group (*p* = 0.674). Subgroup analysis revealed that PSM rates varied by pathological stage: among pT2 cases, margin positivity was 9.1% (7/77) versus 11.2% (9/80) (*p* = 0.674), while among pT3a cases, rates were 25.0% (5/20) versus 41.7% (5/12) (*p* = 0.440), respectively. When stratified by ISUP grade, PSM rates increased with higher grades in both groups: Grade 1 (3.0% vs. 9.4%), Grade 2 (13.2% vs. 17.1%), and Grade 3 (23.1% vs. 21.1%), with no significant differences between techniques within each grade. Similarly, when analyzed by D’Amico risk groups, PSM rates were comparable: Low Risk (3.0% vs. 9.4%), Intermediate Risk (13.2% vs. 17.1%), and High Risk (23.1% vs. 21.1%) (Table 3).

After applying Bonferroni correction for 9 primary outcome comparisons (corrected α = 0.0056), two outcomes remained statistically significant: console time (*p* < 0.001) and time-to-continence recovery (*p* = 0.003). Time-to-potency recovery, while showing a favorable trend for ultrapreservation (*p* = 0.012), did not reach statistical significance after multiple comparison adjustment. All PSM-related outcomes and other perioperative parameters also failed to reach corrected significance levels, though consistent directional effects favored the ultrapreservation technique across multiple domains.

**Figure 3:**
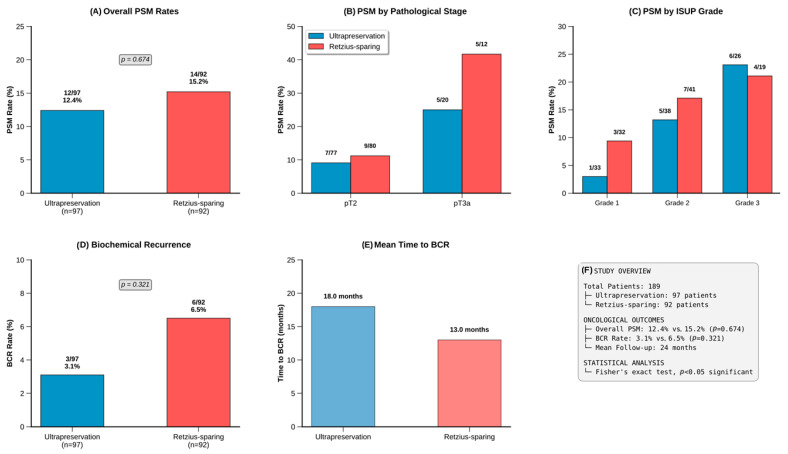
Oncological outcomes comparison between ultrapreservation and Retzius-sparing robotic prostatectomy in 189 patients. (**A**) Overall PSM rates (12.4% vs. 15.2%, *p* = 0.674). (**B**) PSM by pathological stage showing higher rates in pT3a disease. (**C**) PSM by ISUP grade demonstrating increasing rates with higher grades. (**D**) BCR rates (3.1% vs. 6.5%, *p* = 0.321). (**E**) Mean time to BCR (18.0 vs. 13.0 months). (**F**) Study summary with key demographics and outcomes. Statistical analysis: Fisher’s exact test, *p* < 0.05 significant.

Biochemical recurrence occurred in 3.1% of ultrapreservation patients (*n* = 3, mean 18 months) and 6.5% of Retzius-sparing patients (*n* = 6, mean 13 months, *p* = 0.321). No significant difference was observed in biochemical recurrence-free survival (*p* = 0.404) (Figure 3).

### 3.6. Complications

Complications of any grade (≥1) occurred in eight patients (8.2%) in the ultrapreservation group and 10 patients (10.9%) in the Retzius-sparing group. Observed events included urinary tract infection (1 each), surgical site infection (1 each), postoperative nausea/vomiting requiring antiemetics (4 vs. 3), and mild self-limiting hematuria (2 vs. 4). One patient in the Retzius-sparing group required a blood transfusion (1.1%). No Grade ≥ 3 complications were observed (Table 4).

## 4. Discussion

Various techniques for RARP have been described and are routinely applied in clinical practice. The current study is the first to directly compare the ultrapreservation and Retzius-sparing techniques. Our findings demonstrate that both approaches provide oncologically safe outcomes, with the ultrapreservation technique showing advantages in perioperative parameters and potency recovery, while the Retzius-sparing technique offers superior early continence recovery.

The superior perioperative outcomes of the ultrapreservation technique are noteworthy. In our study, the ultrapreservation approach was associated with shorter operative and console times, reduced blood loss, and shorter hospital stays highlight its surgical efficiency. These findings align with recent evidence suggesting that anterior preservation techniques may offer technical advantages due to better anatomical visualization and workspace optimization. We attribute these advantages to the wider working space and clearer anatomical landmarks achieved during lateral pedicle dissection compared with the Retzius-sparing approach [23]. These findings are consistent with previous reports by Kucuk et al. [11].

In radical prostatectomy outcomes, the concept of the “trifecta” includes zero-pad continence as a key measure [24]. Therefore, in our study, we adopted this criterion for the assessment of continence. Our immediate continence results (27.8% ultrapreservation vs. 56.5% Retzius-sparing, *p* < 0.001) are consistent with a recent meta-analysis by Gao et al., which reported immediate continence rates ranging from 51 to 71% for Retzius-sparing approaches compared to 21–48% for standard techniques [8]. In the literature, immediate continence rates following the Retzius-sparing RARP have been reported between 51% and 71%, compared with 21% to 48% for standard or ultrapreservation techniques [25,26]. The superiority of the Retzius-sparing approach in achieving early continence is attributed to the preservation of the Retzius space, puboprostatic ligaments, and endopelvic fascia. This maintains anatomic support around the urethra and bladder neck [12,16].

Systematic reviews and meta-analyses confirm that 12-month continence rates remain higher in Retzius-sparing patients [27]. However, our findings demonstrate that continence in the ultrapreservation group reached comparable levels from the first month onward (93.8% vs. 91.3% at 12 months, *p* = 0.703), demonstrating the long-term effectiveness of both approaches. Literature reports 12-month continence of 95.7% for ultrapreservation RARP, consistent with our findings [11]. Previous studies evaluating the learning curve of Retzius-sparing RARP performed by surgeons experienced in standard RARP have reported 12-month continence rates of 62.8% [28] and 65.7% [14].

Preservation of potency is another key functional outcome following radical prostatectomy [24]. Several studies have demonstrated that sexual dysfunction often causes greater bother and has a more negative impact on patient-reported quality of life than urinary incontinence [29,30]. Our potency recovery rates were notably higher than previously reported, with medium effect sizes observed at 3 and 6 months (Cohen’s h = 0.503 and 0.282, respectively), suggesting clinically meaningful differences favoring ultrapreservation. These findings highlight that optimal potency recovery should be considered not only a functional endpoint but also a critical determinant of overall patient well-being. In the literature, 12-month potency rates after ultrapreservation RARP have been reported as 67.4% [11]. In our study, 12-month potency recovery rates were at the upper end of this range in both groups (82.1% vs. 72.5%). We attribute the higher postoperative potency rates observed in our study, compared with previous reports, to the growing experience of our center with this technique over time.

The 14-day difference in median continence recovery time (28 vs. 42 days) between ultrapreservation and Retzius-sparing techniques represents a clinically meaningful advantage. This earlier recovery translates to reduced patient anxiety, improved quality of life, and decreased healthcare resource utilization during the critical early postoperative period. The hazard ratio of 1.65 indicates that patients undergoing ultrapreservation have a 65% higher likelihood of achieving continence at any given time point, providing a robust measure of the technique’s benefit. Similarly, while the 42-day difference in median potency recovery time (84 vs. 126 days) did not reach statistical significance after multiple comparison adjustment, the hazard ratio of 1.42 suggests a clinically relevant advantage that may become statistically apparent in larger studies. The consistent directional benefit across both functional outcomes supports the biological plausibility of superior anatomical preservation with the ultrapreservation approach.

The significantly higher rate of bilateral nerve-sparing in the ultrapreservation group (83.5% vs. 70.7%, *p* = 0.034) represents both a strength and potential confounding factor in our potency analysis. This difference may reflect improved surgical confidence and anatomical preservation capabilities of the ultrapreservation technique, allowing for more aggressive nerve preservation. However, it also introduces a potential bias favoring potency outcomes in the ultrapreservation group, as bilateral nerve-sparing is a well-established predictor of erectile function recovery following radical prostatectomy.

The non-significant findings for several secondary outcomes should be interpreted within the context of statistical power limitations. While our study achieved excellent power (>98%) for the primary endpoint, secondary outcomes were not formally powered during study design. This is particularly relevant for potency recovery, where a 9.6 percentage point difference (82.1% vs. 72.5%) at 12 months, while not statistically significant (*p* = 0.214), may represent a clinically meaningful advantage that warrants validation in larger, adequately powered studies.

The most important oncological outcomes following radical prostatectomy include positive surgical margins (PSM) and biochemical recurrence. Our subgroup analysis revealed important patterns: PSM rates increased with pathological stage (pT2: 9.1% vs. 11.2%; pT3a: 25.0% vs. 41.7%) and ISUP grade (Grade 1: 3.0% vs. 9.4%; Grade 3: 23.1% vs. 21.1%), consistent with established risk factors. These findings align with a recent meta-analysis reporting higher PSM rates in Retzius-sparing approaches, particularly for ≥pT3 tumors (OR = 1.81, 95% CI = [1.18–2.77], *p* = 0.006) [8]. A meta-analysis by Novara et al. reported PSM rates of 4–23% in pT2 cancers and 29–50% in pT3 cancers [31]. The data from our series are consistent with these findings in the literature, indicating that both techniques are oncologically safe. The comparable oncological outcomes suggest that the ultrapreservation technique provides functional advantages without compromising cancer control.

The 22-month mean follow-up duration, while providing robust data for early functional recovery comparison, represents a limitation for definitive oncological conclusions. The comparable biochemical recurrence rates observed between groups (3.1% vs. 6.5%, *p* = 0.321) should be interpreted cautiously, as the median time to biochemical recurrence following radical prostatectomy typically ranges from 24 to 36 months in contemporary series [32,33]. Extended follow-up will be essential to validate the oncological equivalence suggested by our intermediate-term results.

Furthermore, the functional outcome advantages observed with each technique may evolve over time. While our 12-month functional assessments demonstrate clear patterns, some patients experience continued functional improvement beyond the first postoperative year, warranting long-term follow-up studies.

The retrospective design of this study represents the most significant limitation, introducing potential selection bias and confounding variables that may influence outcomes. Single-surgeon experience, while reducing surgical variability, may limit generalizability to other centers and surgeons. The patient allocation criteria were not randomized, potentially introducing selection bias based on surgeon preference, patient anatomy, or temporal factors. Additionally, the study period (2022–2024) coincides with the initial description of ultrapreservation technique in 2023, raising questions about potential learning curve effects on early cases.

The absence of validated questionnaires such as the International Index of Erectile Function-5 (IIEF-5) for potency assessment and the International Consultation on Incontinence Questionnaire-Urinary Incontinence Short Form (ICIQ-UI) for continence evaluation represents a limitation in functional outcome assessment. Future studies should incorporate these validated tools to enhance the clinical applicability of findings.

This study addressed several methodological limitations through comprehensive effect size calculations and subgroup analyses. The large effect size for console time (Cohen’s d = −1.188) and medium effect sizes for immediate continence recovery (Cohen’s h = −0.590) and early potency recovery (Cohen’s h = 0.503) suggest clinically meaningful differences between techniques. These effect sizes provide important context for clinical decision-making and future study design.

This study was specifically powered for the primary endpoint of immediate continence recovery. Secondary outcomes including long-term potency recovery, positive surgical margins, and complication rates were not subject to formal power calculations during study design. Consequently, negative findings for these endpoints should be interpreted cautiously, as they may reflect Type II errors (false negatives) rather than true equivalence between techniques.

The observed non-significant differences in 12-month potency recovery (82.1% vs. 72.5%, *p* = 0.214) and positive surgical margin rates (12.4% vs. 15.2%, *p* = 0.674) may represent clinically meaningful differences that our study lacked adequate power to detect. Future studies should incorporate formal power calculations for all clinically relevant endpoints to ensure adequate sample sizes for comprehensive comparisons.

This study provides valuable preliminary data for the design of future randomized controlled trials comparing ultrapreservation and Retzius-sparing techniques. Based on our findings, adequately powered multicenter studies should incorporate validated questionnaires (IIEF-5, ICIQ-UI), longer follow-up periods (≥24 months), and propensity score matching to control for confounding variables. Additionally, learning curve analyses and cost-effectiveness evaluations would enhance clinical translation of these findings.

Despite these limitations, our study makes a meaningful contribution to the literature as the first direct comparison between the ultrapreservation and Retzius-sparing techniques, providing effect size calculations and comprehensive subgroup analyses that inform clinical practice.

## 5. Conclusions

This is the first study to directly compare the ultrapreservation and Retzius-sparing techniques. Both approaches offer unique advantages: the ultrapreservation technique provides superior perioperative outcomes and potency recovery with large to medium effect sizes (Cohen’s d = −1.188 for console time, Cohen’s h = 0.503 for early potency recovery), whereas the Retzius-sparing technique offers earlier continence recovery with medium effect size (Cohen’s h = −0.590). Both techniques yield acceptable oncological outcomes with comparable PSM rates across D’Amico risk groups and pathological stages.

**Figure 4:**
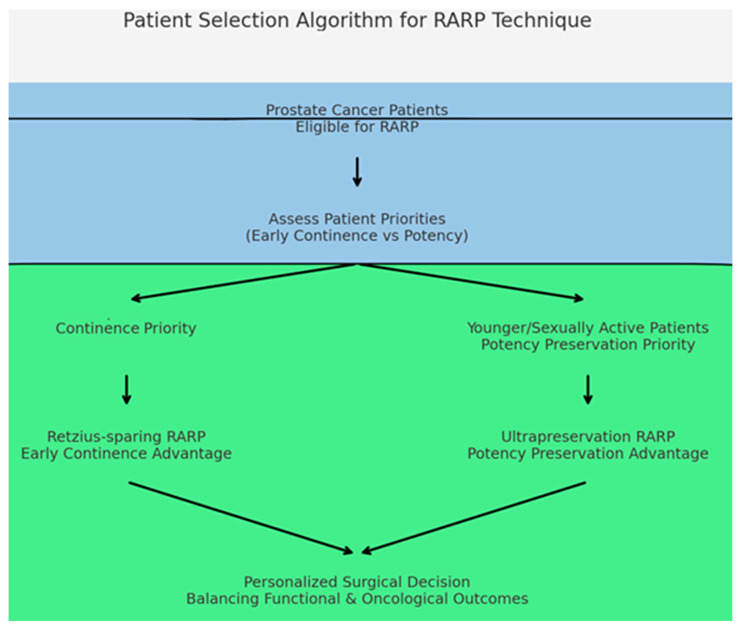
Proposed Patient Selection Algorithm for Robotic Radical Prostatectomy Technique Choice.

Surgical technique selection should be individualized based on patient characteristics, surgeon experience, and patient priorities. Ultrapreservation may be preferred in younger patients with good preoperative potency, while Retzius-sparing may be considered when early continence recovery is the primary goal (Figure 4). Further research, including randomized controlled trials with larger patient populations and validated questionnaires (IIEF-5, ICIQ-UI), is warranted to validate these findings.

Clinical decision-making flowchart incorporating patient age, baseline erectile function status, continence priorities, and individual preferences. The algorithm based on the study findings demonstrated complementary advantages: ultrapreservation for younger patients with preserved baseline potency prioritizing erectile function recovery; Retzius-sparing for patients prioritizing immediate continence recovery. Final decisions should incorporate shared decision-making principles and individual patient values. Sample sizes: Ultrapreservation *n* = 97; Retzius-sparing *n* = 92.

## Figures and Tables

**Table 1:** Demographic and clinical characteristics.

Variable		Ultrapreservation (*n* = 97)	Retzius-Sparing (*n* = 92)	*p*-Value
Age (years), mean (SD)		61.5 (3.1)	60.9 (3.2)	0.2
BMI (kg/m^2^), mean (SD)		26.3 (2.1)	26.0 (2.0)	0.353
Charlson Comorbidity Index		2.0 ± 0.7	2.4 ± 0.6	**<0.001**
Pre-operative Potency, *n* (%)		78 (80.4)	80 (87.0)	0.309
PSA (ng/mL), mean (SD)		6.8 (1.4)	6.6 (1.6)	0.438
Prostate Volume (mL), mean (SD)		45.8 (7.0)	44.9 (8.1)	0.436
D’Amico Risk Group, *n* (%)				0.581
Low Risk		33 (34.0)	32 (34.8)	
Intermediate Risk		38 (39.2)	41 (44.6)	
High Risk		26 (26.8)	19 (20.7)	
ISUP Grade Group, *n* (%)	1	33 (34.0)	32 (34.8)	0.581
	2	38 (39.2)	41 (44.6)	
	3	26 (26.8)	19 (20.7)	
Pathologic Stage, *n* (%)	pT2	77 (79.4)	80 (87.0)	0.233
	pT3a	20 (20.6)	12 (13.0)	
Nerve Sparing, *n* (%)	None	5 (5.2)	12 (13.0)	0.077
	Unilateral	11 (11.3)	15 (16.3)	
	Bilateral	81 (83.5)	65 (70.7)	
Follow-up, months, mean (range)		22.0 (14–32)	22.9 (14–36)	0.196

BMI: body mass index, ISUP: International Society of Urological Pathology, PSA: prostate-specific antigen. Significant *p*-values are written in bold. D’Amico Risk Classification: Low risk (PSA ≤ 10 ng/mL, Gleason score ≤ 6, and clinical stage ≤ T2a), intermediate risk (PSA 10.1–20 ng/mL, Gleason score 7, or clinical stage T2b), and high risk (PSA > 20 ng/mL, Gleason score ≥ 8, or clinical stage ≥ T2c).

**Table 2:** Perioperative outcomes.

Variable	Ultrapreservation (*n* = 97)	Retzius-Sparing (*n* = 92)	*p*-Value
Operative time (min), mean (SD)	174.8 (19.5)	188.7 (14.9)	**<0.001**
Console time (min), mean (SD)	112.4 (14.3)	132.0 (18.5)	**<0.001**
Blood loss (mL), mean (SD)	119.0 (30.7)	133.3 (33.4)	**0.002**
Hospital stay (days), mean (SD)	2.3 (0.5)	2.5 (0.6)	**0.004**

Significant *p*-values are written in bold.

**Table 3:** Comprehensive positive surgical margin analysis with risk stratification.

Variable	Ultrapreservation (*n* = 97)	Retzius-Sparing (*n* = 92)	*p*-Value
Overall PSM, *n* (%)	12 (12.4)	14 (15.2)	0.674
PSM by Pathologic Stage			
pT2 (*n* = 157)	7/77 (9.1)	9/80 (11.2)	0.674
pT3a (*n* = 32)	5/20 (25.0)	5/12 (41.7)	0.440
PSM by ISUP Grade			
Grade 1 (*n* = 65)	1/33 (3.0)	3/32 (9.4)	0.349
Grade 2 (*n* = 79)	5/38 (13.2)	7/41 (17.1)	0.771
Grade 3 (*n* = 45)	6/26 (23.1)	4/19 (21.1)	1.000
PSM by D’Amico Risk Group			
Low Risk (*n* = 65)	1/33 (3.0)	3/32 (9.4)	0.349
Intermediate Risk (*n* = 79)	5/38 (13.2)	7/41 (17.1)	0.771
High Risk (*n* = 45)	6/26 (23.1)	4/19 (21.1)	1.000

PSM: Positive surgical margin, ISUP: International Society of Urological Pathology.

**Table 4:** Postoperative complications classified by Clavien-Dindo system.

Clavien-Dindo	Ultrapreservation	Retzius-Sparing
Grade 0 (No Complication)	89 (91.8%)	82 (89.13%)
Grade 1 (Minor)	6 (6.2%)	7 (7.6%)
Grade 2	2 (2.1%)	3 (3.26%)
Grade ≥ 3 (Major)	0 (0.0%)	0 (0.0%)

## Data Availability

The raw data supporting the conclusions of this article are not publicly available due to patient privacy and ethical considerations. Anonymized aggregate datasets and statistical analysis protocols are available from the corresponding author upon reasonable request to qualified researchers with appropriate institutional ethical approval for secondary analysis purposes.

## Abbreviations

The following abbreviations are used in this manuscript:

BMI	Body Mass Index
CI	Confidence Interval
DVC	Dorsal Venous Complex
HR	Hazard Ratio
ICIQ-UI	International Consultation on Incontinence Questionnaire-Urinary Incontinence Short Form
IIEF-5	International Index of Erectile Function-5
IPSS	International Prostate Symptom Score
ISUP	International Society of Urological Pathology
NVBs	Neurovascular Bundles
PSA	Prostate-Specific Antigen
PSM	Positive Surgical Margin
RARP	Robotic-Assisted Radical Prostatectomy
SD	Standard Deviation
SHIM	Sexual Health Inventory for Men
STROBE	Strengthening the Reporting of Observational Studies in Epidemiology
